# Establishment of a relationship between blastomere geometry and YAP localisation during compaction

**DOI:** 10.1242/dev.189449

**Published:** 2020-10-09

**Authors:** Christophe Royer, Karolis Leonavicius, Annemarie Kip, Deborah Fortin, Kirtirupa Nandi, Anna Vincent, Celine Jones, Tim Child, Kevin Coward, Chris Graham, Shankar Srinivas

**Affiliations:** 1 Department of Physiology, Anatomy and Genetics, University of Oxford, Oxford OX1 3QX, UK; 2 Oxford Fertility, Institute of Reproductive Sciences, Oxford OX4 2HW, UK; 3 Nuffield Department of Women's and Reproductive Health, University of Oxford, Oxford OX3 9DU, UK

**Keywords:** Biocompatible polymers, Compaction, Hippo signalling, Human embryo

## Abstract

Precise patterning within the three-dimensional context of tissues, organs and embryos implies that cells can sense their relative position. During preimplantation development, outside and inside cells rely on apicobasal polarity and the Hippo pathway to choose their fate. Despite recent findings suggesting that mechanosensing might be central to this process, the relationship between blastomere geometry (i.e. shape and position) and the Hippo pathway effector YAP remains unknown. We used a highly quantitative approach to analyse information on the geometry and YAP localisation of individual blastomeres of mouse and human embryos. We identified the proportion of exposed cell surface area as most closely correlating with the nuclear localisation of YAP. To test this relationship, we developed several hydrogel-based approaches to alter blastomere geometry in cultured embryos. Unbiased clustering analyses of blastomeres from such embryos revealed that this relationship emerged during compaction. Our results therefore pinpoint the time during early embryogenesis when cells acquire the ability to sense changes in geometry and provide a new framework for how cells might integrate signals from different membrane domains to assess their relative position within the embryo.

## Abstract (precis)

**Highlighted Article:** Localisation of YAP, a key factor during the first cell fate decision, is linked to individual blastomere geometry within the three-dimentional environment of the preimplantation embryo.

## INTRODUCTION

Through the course of embryonic development, complex tissues and organs are formed from a single cell. This requires each given cell within the developing embryo constantly to sense its position within its three-dimensional environment in order to make correct fate decisions. Morphogen gradients have long been suggested to play an important role in transmitting positional information across tissues during embryo patterning (Briscoe and Small, 2015; Tickle et al., 1975), although even here, mechanistic details remain unclear (Wolpert, 2016). When small groups of cells are concerned, morphogen gradients are conceptually harder to set up, leading cells to rely on other strategies. In the mouse preimplantation embryo, around the 16-cell stage, blastomeres differentiate into trophectoderm (TE) or inner cell mass (ICM) cells depending on their outside or inside position, respectively. The molecular mechanisms by which outside and inside cells determine their fate has been studied extensively, highlighting the importance of apicobasal polarity and Hippo signalling (Plusa et al., 2005; Sasaki, 2017; White et al., 2018).

In apolar inner cells, AMOT localises at cell-cell junctions, where it associates with NF2 and is phosphorylated by the Hippo pathway kinases LATS1/2 (Cockburn et al., 2013; Hirate et al., 2013). In concert with LATS1/2, AMOT is then able to induce the phosphorylation of YAP and its sequestration to the cytoplasm. In inner cells, YAP is consequently unable to bind to TEAD4 and cannot induce the transcription of TE genes, such as *CDX2* (Nishioka et al., 2007, 2009). Instead, genes associated with pluripotency, such as *SOX2*, drive ICM fate in these cells (Wicklow et al., 2014). In outer cells, the establishment of apicobasal polarity generates a contact-free membrane domain where cortical F-actin sequesters AMOT away from cell-cell junctions, preventing its phosphorylation and interaction with Hippo pathway components (Hirate and Sasaki, 2014). This results in the inability of the Hippo pathway to phosphorylate YAP, which can then translocate to the nucleus and interact with TEAD4 to drive the expression of TE-specific genes, such as *CDX2*, consequently inducing the TE fate. Together, this body of work, in addition to highlighting the importance of the Hippo pathway and apicobasal polarity in the first cell fate decision, also suggests multiple links between cytoskeletal organisation and Hippo pathway regulation.

It is now widely accepted that forces related to changes in cell shape or cell position within a group of cells can modulate the localisation and activity of YAP via the actin cytoskeleton (Aragona et al., 2013; Dupont et al., 2011; Halder et al., 2012; Wada et al., 2011). In the preimplantation embryo, although it has been suggested that mechanosensing might occur (Maître et al., 2016) and despite the multiple links between the Hippo pathway and the actin cytoskeleton, it remains unclear whether the shape or position of individual blastomeres can regulate the subcellular localisation of YAP directly to modulate cell fate.

To answer this question, we generated a collection of embryos from the two- to the 64-cell stage, quantifying the subcellular localisation of YAP in addition to several descriptors of blastomere shape and position. When considering blastomeres across all these embryonic stages, we found a progressive increase in the proportion of blastomeres with higher nuclear versus cytoplasmic YAP. We used a multivariate analysis on this dataset to test whether any of the quantitative descriptors of blastomere shape and position showed a correlation with the ratio of nuclear to cytoplasmic YAP (N/C YAP ratio). This revealed that the proportion of exposed surface area of a blastomere had the strongest correlation with differences in YAP localization. Using non-invasive methods to modulate the shape and position of blastomeres within embryos, we demonstrated that this relationship emerged as early as the eight-cell stage, during compaction.

## RESULTS

### Cells with a high N/C YAP ratio occur before the first cell fate decision

**Fig. 1.**
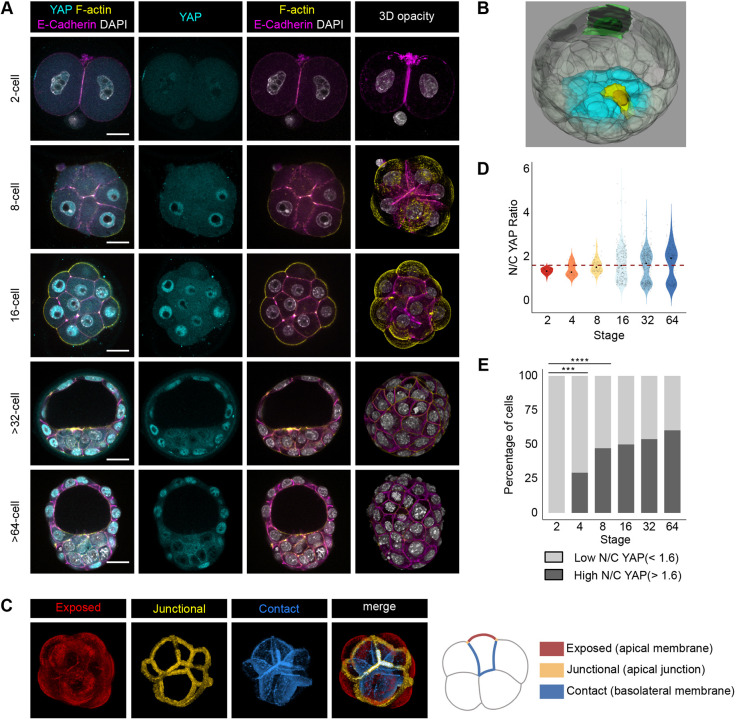
**Analysis of** **the** **N/C YAP ratio across preimplantation development using manual segmentation.** (A) Immunostaining of preimplantation embryos using antibodies against YAP and E-cadherin. F-actin and nuclei were visualised using Phalloidin and DAPI, respectively. (B) Example of a manually segmented 32-cell blastocyst showing blastomeres (green and yellow cells) exhibiting different shapes. Part of the cells making the trophectoderm are not displayed, in order to be able to see inside the blastocyst cavity. The ICM is highlighted in cyan. (C) Blastomere membranes were segmented to obtain blastomere ‘exposed’, ‘junctional’ and ‘contact’ surfaces corresponding to the apical membrane, apical junction and basolateral membrane, respectively. (D) Representation of the relative amount of YAP in the nucleus and cytoplasm (N/C YAP ratio) of individual blastomeres at the two- (*n*=20 embryos), four- (*n*=8 embryos), eight- (*n*=10 embryos), 16- (*n*=17 embryos), 32- (*n*=12 embryos) and 64-cell stage (*n*=2 embryos). A black dot indicates the median N/C YAP ratio for each developmental stage. (E) Representation of the proportion of blastomeres with low and high N/C YAP ratio across developmental stages. Blastomeres from all stages were classified as exhibiting either a high (>1.6) or a low (<1.6) N/C YAP ratio based on a *k*-means algorithm to separate them into two populations in an unbiased manner. The threshold is also represented in D by a dashed line. Scale bars: 20 µm. ***P<0.001, ****P<0.0001 (Fisher's exact test).

In a two-dimensional environment, cellular parameters such as shape and position, which we refer to as cell geometry, have been shown to affect the localisation (and therefore activity) of YAP through its mechanosensing properties (Aragona et al., 2013; Dupont et al., 2011; Wada et al., 2011). In the preimplantation embryo, despite the suggestion that mechanosensing might be involved in the regulation of YAP activity (Maître et al., 2016), it remains unclear whether geometrical properties of blastomeres directly influence the relative distribution of YAP to the nucleus and cytoplasm. To analyse the relationship between the localisation of YAP and the geometry of individual blastomeres during mammalian preimplantation cell fate allocation, we used a highly quantitative approach combining imaging, manual segmentation and image analysis. We collected and stained mouse embryos for YAP, F-actin and E-cadherin at the two- (*n*=20), four- (*n*=8), eight- (*n*=10), 16- (*n*=17), 32- (*n*=12) and 64-cell (*n*=2) stages (Fig. 1A; Fig. S1A).

We next created three-dimensional cellular-resolution volume representations of these embryos by manually segmenting the cell membrane and nucleus of individual blastomeres (Fig. 1B; Movie 1). This allowed us to quantify the cellular localisation of YAP accurately in the nuclear and cytosolic compartments by determining the N/C YAP ratio. We were also able to quantify the area of the blastomere limiting membrane and categorise it as ‘exposed to the outside’, ‘in contact with other blastomeres’ or ‘junctional’ (Fig. 1C) (Leonavicius et al., 2020). This allowed us to quantify three parameters of blastomere shape [sphericity, oblateness (flying saucer shaped) and prolateness (rugby ball shaped)], two parameters relating to blastomere position (proportion of surface exposed to outside and proportion of surface in contact with other blastomeres) and six parameters relating to other aspects of blastomere geometry (see Materials and Methods for a description of all parameters and how they were calculated).

When we considered all blastomeres at each stage, we found that, as expected, two populations of blastomeres with distinct N/C YAP ratios seemed to appear progressively from the 16-cell stage onwards (Fig. 1D). In order to test this observation quantitatively, we used *k*-means clustering of N/C YAP values of all the blastomeres in our dataset, spanning the two- to 64-cell stage, to separate cells with high versus low N/C YAP ratio in an unbiased manner. This clustering found a N/C YAP ratio of 1.6 as the threshold separating ‘low’ and ‘high’ nuclear YAP cells, which would be expected to correspond to inside (or ICM) and outside (or TE) cells, respectively. To test independently the validity of this threshold in categorising cells, we classified cells from 32- and 64-cell embryos manually as ICM or TE depending on their position. We found that the vast majority of TE cells were correctly classified as having a high N/C YAP ratio (111/116, 95.7%), and all ICM cells were classified as having a low N/C YAP ratio (Fig. S1B). Using this classification, we observed that, as published before when comparing inside and outside cells, these two populations become strongly separate from the 16-cell stage onwards (Fig. S1C) (Hirate et al., 2015; Nishioka et al., 2009), thereby validating the representativeness of our dataset. However, our detailed quantification also revealed that an increasing proportion of cells with a high N/C YAP ratio arises from the four- to the 16-cell stage, suggesting that cells with a relatively high N/C YAP ratio exist even before the 16-cell stage and the establishment of distinct outside and inside cells (Fig. 1D; Fig. S1C).

### Proportion of exposed surface, as opposed to shape, is strongly associated with N/C YAP ratio

To determine whether geometrical properties of blastomeres might influence the relative distribution of YAP to the nucleus and cytoplasm directly, we interrogated our quantitative dataset for the correlation between the N/C YAP ratio and the various parameters relating to blastomere shape and position that we had extracted.

**Fig. 2.**
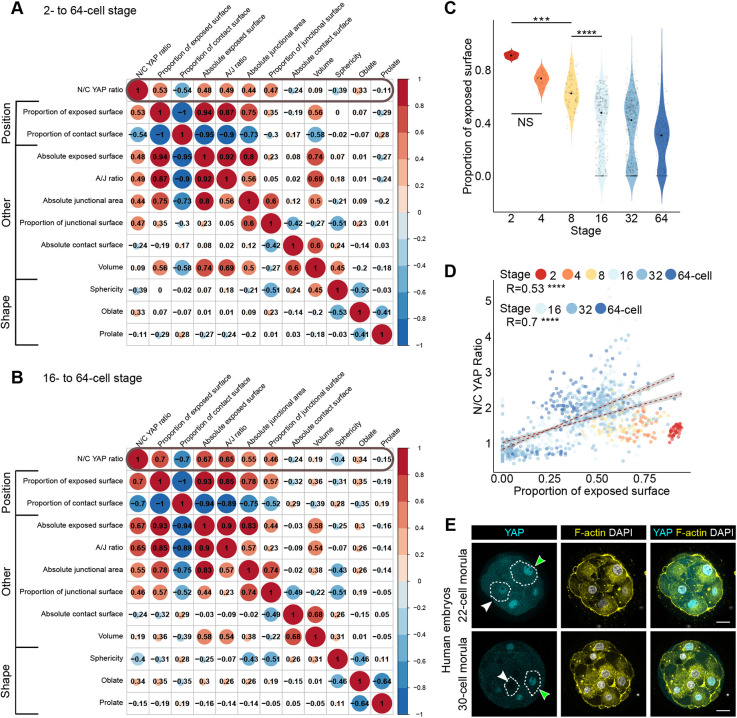
**The proportion of exposed surface is associated with the proportion of YAP in the nucleus.** (A) Correlation matrix between N/C YAP ratio and geometric characteristics of individual blastomeres across preimplantation development. (B) Correlation matrix between N/C YAP ratio and geometric characteristics of individual blastomeres from the 16- to the 64-cell stage. Note how the proportion of exposed surface and its converse, the proportion of contact surface, are correlated the highest with the N/C YAP ratio. In A and B, the value of the correlation coefficient (Spearman) between two variables is indicated and also represented by the size and colour of the circles. (C) Proportion of exposed blastomere surface area across developmental stages. The median proportion of exposed surface area for each developmental stage is represented as a black dot. NS=not significant, **P<0.01, ***P<0.001 (Kruskal–Wallis test followed by Dunn's test). (D) Correlation analysis between the proportion of exposed cell surface area (an indicator of position) and the N/C YAP ratio at the indicated stages. ****P<0.0001 (Spearman). (E) Representative optical sections of human morulae containing the indicated number of cells and immunostained for YAP. White arrowheads indicate cells with either low or no exposed cell surface area and low nuclear YAP, whereas green arrowheads indicate cells with high exposed cell surface area and high nuclear YAP. F-actin and nuclei were visualised using Phalloidin and DAPI, respectively. Scale bars: 20 µm.

Blastomere shape parameters (sphericity, oblateness and prolateness) exhibited relatively poor correlations with N/C YAP ratio (Fig. 2A,B). However, of the three blastomere shape descriptors, sphericity showed the highest correlation (Fig. 2A,B). Sphericity decreased gradually from the two- to the 64-cell stage, most probably because of the increasingly varied blastomere shapes arising as the embryos developed (Fig. S2A). The N/C YAP ratio and sphericity showed a weak negative correlation (*R*=−0.39; *P*<2.2×10^−16^) when considering all blastomeres from the two- to the 64-cell stage (Fig. S2B). This correlation became slightly stronger if only blastomeres of the 32- and 64-cell stages were considered (i.e. as outside cells become more stretched and elongated in shape), but still remained relatively weak (*R*=−0.54; *P*<2.2×10^−16^) (Fig. S2B). Together, these data show that blastomere shape is a relatively poor predictor of YAP localisation, suggesting that it is unlikely that overall cell shape would modulate YAP localisation directly.

On the other hand, position parameters, such as proportion of exposed surface area and its converse, proportion of contact area, showed the strongest correlation with the N/C YAP ratio, both across our entire dataset and more so when considering the subset from the 16- to 64-cell stage. Interestingly, the N/C YAP ratio and the A/J ratio (the ratio between blastomere apical surface area and apical junctional interface area, estimating the extent to which the apical surface protrudes outwards) were also closely correlated, suggesting that the shape of the apical domain might be linked to the proportion of YAP in the nucleus and cytoplasm (Fig. 2A,B; Fig. S2C). Interestingly, at the 16-cell stage, the N/C YAP ratio was strongly correlated with the proportion of exposed surface and contact areas, the A/J ratio and the absolute exposed blastomere surface area (Fig. S2D). Unsurprisingly, we found that overall, the proportion of exposed cell surface area decreased continuously from the two- to 64-cell stage, in line with the fact that, as the ICM forms, more cells end up inside. We also saw a dramatic decrease in the overall proportion of exposed cell surface area from the two- to eight-cell stage, which might be linked to the process of compaction (Fig. 2C). When examining the relationship between the proportion of exposed cell surface area and the N/C YAP ratio, considering blastomeres across all stages, we found that they were only moderately correlated (*R*=0.57; *P*<2.2×10^−16^). However, this association became strong when considering blastomeres from the 16-cell stage onwards (*R*=0.7; *P*<2.2×10^−16^) (Fig. 2D).

To determine whether a similar relationship existed in human embryos, we examined the localisation of YAP in human compacted morulae. We found that blastomeres with a high proportion of exposed surface area exhibited high levels of YAP in their nuclei, whereas cells on the outside that were more embedded within the embryo had reduced nuclear YAP and completely inside cells had close to no nuclear YAP. This suggests that cell fate allocation in the human follows the same principles as those during mouse preimplantation development (Fig. 2E). Together, our findings suggest that the amount of YAP in the nucleus is proportional to the extent to which cells are outside or inside, based on the proportion of exposed surface. This suggests that the molecular mechanism acting to regulate the localisation of YAP is able to sense the relative amount of exposed cell surface area very accurately.

### The relationship between the proportion of exposed surface and the N/C YAP ratio is established during compaction

**Fig. 3.**
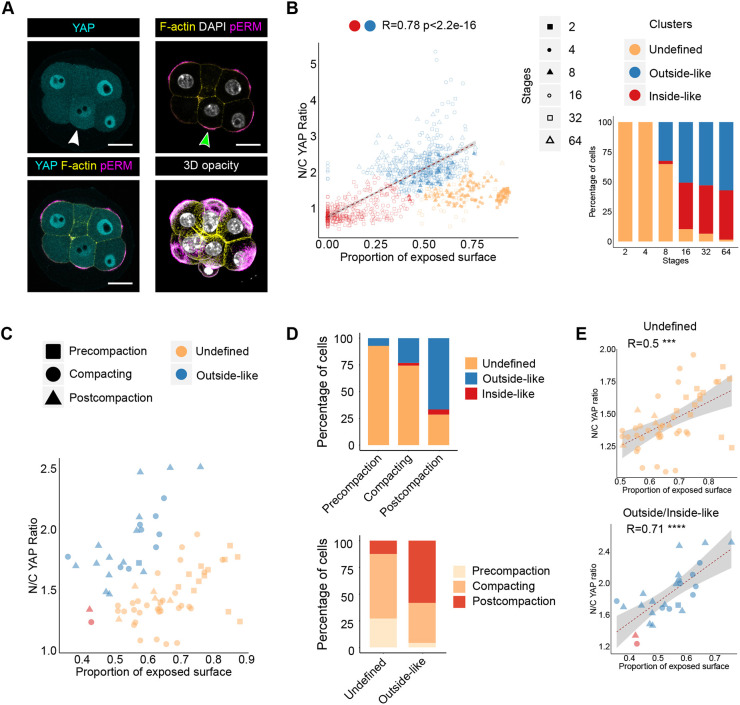
**Hierarchical clustering analysis reveals the association between the proportion of exposed surface and the N/C YAP ratio in compacted eight-cell embryos.** (A) Images of an eight-cell embryo immunostained for YAP and pERM, illustrating variations in the N/C YAP ratio at the eight-cell stage. F-actin and nuclei were visualised using Phalloidin and DAPI, respectively. White arrowhead indicates a blastomere with lower N/C YAP ratio. Green arrowhead highlights the presence of apical pERM. Bottom right panel shows a three-dimensional (3D) opacity rendering of the corresponding embryo. Scale bars: 20 µm. (B) Hierarchical clustering of blastomeres across preimplantation development into three distinct clusters. Blastomeres with a high N/C YAP ratio and intermediate proportion of exposed cell surface area were classified as belonging to the outside-like cluster. Blastomeres with a low N/C YAP ratio and low exposed cell surface area were classified as belonging to the inside-like cluster. Finally, the remaining blastomeres, exhibiting a high proportion of exposed cell surface area and intermediate N/C YAP ratio were defined as belonging to an ‘undefined cluster’. Dot shape indicates stage, whereas colour indicates the cluster to which each blastomere belongs (Spearman, *R*=0.78, *P*<2.2×10^−16^). Bottom right bar graph represents the distribution of blastomeres across the three clusters for each stage. (C) Analysis of the N/C YAP ratio and the proportion of exposed cell surface area at the eight-cell stage in precompaction, compacting and postcompaction embryos. (D) Bar graphs representing the proportion of blastomeres from precompaction, compacting and postcompaction embryos in the undefined, inside-like and outside-like clusters (top). The proportion of blastomeres from each cluster found in precompaction, compacting and postcompaction embryos is shown at the bottom. (E) Correlation between the proportion of exposed surface and the N/C YAP ratio in undefined (top) (Spearman, *R*=0.52, *P*=1.4×10^−4^) and inside- and outside-like eight-cell blastomeres (bottom) (Spearman, *R*=0.71, *P*=5.3×10^−5^).

Although the relationship between the proportion of exposed cell surface area and the N/C YAP ratio became strong from the 16-cell stage onwards, it was sometimes possible to observe blastomeres at the eight-cell stage that already had a lower amount of YAP in the nucleus and also appeared more deeply embedded within the embryo (Fig. 3A). Furthermore, with respect to the relationship between the N/C YAP ratio and the proportion of exposed surface area, some blastomeres from eight-cell embryos fell into the region of the plot occupied by blastomeres from later stage embryos, where a stronger relationship exists between these two parameters (Fig. 2D). This suggests that blastomeres might already be able to sense their position through the proportion of their surface exposed at the eight-cell stage.

To test whether there might be blastomeres at the eight-cell stage that already display a relationship between the N/C YAP ratio and the proportion of exposed cell surface area, we first tested whether blastomeres across development could be classified according to only these two characteristics. To do this, we performed unsupervised hierarchical clustering of our entire dataset using the N/C YAP ratio and the proportion of exposed cell surface area as variables and found that on the basis of only these two parameters, blastomeres could be grouped into three distinct clusters (Fig. 3B). One was characterised by a low N/C YAP ratio and a low proportion of exposed surface area, a second by a high N/C YAP ratio and an intermediate proportion of exposed surface area and the third by an intermediate N/C YAP ratio and a high proportion of exposed surface area (Fig. S3A-C). Based on these observations, blastomeres from the first two clusters exhibited ICM- and TE-like characteristics, respectively, whereas the third cluster potentially represented blastomeres in an ‘undecided’ state, because it contained large numbers of blastomeres from the two-, four- and eight-cell stages. We therefore named the clusters ‘inside-like’, ‘outside-like’ and ‘undefined’, respectively. Consistent with developmental trajectories, we could detect a strong positive correlation between the N/C YAP ratio and the proportion of exposed surface when only the outside-like and inside-like clusters were considered together, without the undefined cluster (Fig. 3B).

To determine how well the unsupervised clustering performed, we next categorised blastomeres from a subset of 32- and 64-cell stage embryos manually as inside or outside, to generate a ground truth against which to compare the unbiased clustering. We found that all blastomeres annotated manually as ICM fell into the inside-like cluster (82/82), and the vast majority annotated as TE fell into the outside-like cluster (108/116, 93.1%). Conversely, the outside-like cluster consisted exclusively of TE cells (109/109), whereas the inside-like cluster consisted almost exclusively of ICM cells (82/88, 93.2%). Overall, the clustering displayed very few errors, and these lay at the interface of the different clusters (Fig. S3D). The broad accuracy of the unsupervised clustering suggests that the blastomeres clustering as ‘undefined’ might represent a biologically meaningful state.

All two- and four-cell stage blastomeres belonged to the undefined cluster, whereas only two-thirds of those from the eight-cell stage belonged to this cluster, with the remaining one-third falling in the outside-like cluster (Fig. 3B). Thereafter, a much lower proportion of 16- to 64-cell stage blastomeres belonged to the undefined cluster (Fig. 3B; Fig. S3E). Taken together, this again suggested that at the eight-cell stage, differences were already starting to emerge amongst blastomeres, with those first to become different being on a TE trajectory, and that ICM cells mostly arise only at the 16-cell stage, when cells start being found inside the embryo.

To verify whether blastomeres at the eight-cell stage have already acquired the ability to sense and respond to their position and whether this is linked to embryo maturation, we examined eight-cell embryos at different stages of compaction in more detail (Fig. 3C). We categorised eight-cell embryos based on their morphology as ‘precompaction’, ‘compacting’ and ‘postcompaction’. In validation of our categorisation, the morphometric parameters of blastomere sphericity and proportion of exposed cell surface were significantly different in postcompaction embryos (Fig. S3F,G).

Interestingly, the majority of blastomeres from postcompaction eight-cell embryos belonged to the outside-like cluster (14/21, 66.7%). By contrast, all but one blastomere from precompaction eight-cell embryos belonged to the undefined cluster (13/14, 92.9%) (Fig. 3D). The allocation of most blastomeres from postcompaction embryos to the outside-like cluster and from precompaction embryos to the inside-like cluster could not be explained by changes in YAP localisation alone, because there were no significant differences in the N/C YAP ratio between blastomeres depending on the degree of embryo compaction (Fig. S3H). Amongst blastomeres from the undefined cluster, there was only a weak relationship between the proportion of exposed surface area and the N/C YAP ratio (Fig. 3E), suggesting that these blastomeres might not yet be able to sense the proportion of cell membrane exposed to the outside. By contrast, eight-cell blastomeres from the outside-like and inside-like clusters, which came predominantly from postcompaction embryos, exhibited a strong relationship between the proportion of exposed cell surface area and the N/C YAP ratio (Fig. 3E). This indicates that the relationship between the proportion of exposed surface and the N/C YAP ratio emerges during compaction, suggesting that the position-sensing machinery might be in place earlier than previously thought, by the time compaction is completed.

### Increase in N/C YAP ratio from the two- to eight-cell stage is dependent on biochemical changes occurring during compaction

Given that our results suggest that the ability of blastomeres to sense the relative amount of their surface area exposed is acquired progressively during compaction, we sought to perturb the biochemical events taking place during compaction using Ro-31-8220 (RO), an inhibitor of protein kinase C (PKC) and other kinases. Treatment of eight-cell embryos with RO led to a reduced phosphorylation of ERM proteins (pERM), probably through the inhibition of aPKC subtypes (Liu et al., 2013). This, in turn, was shown to lead to decreased apical F-actin (Liu et al., 2013), which would be expected to impair some of the morphogenic process associated with compaction in addition to the establishment of blastomere polarity, because the actomyosin network is required for the apical localisation of the PAR complex (Zhu et al., 2017).

**Fig. 4.**
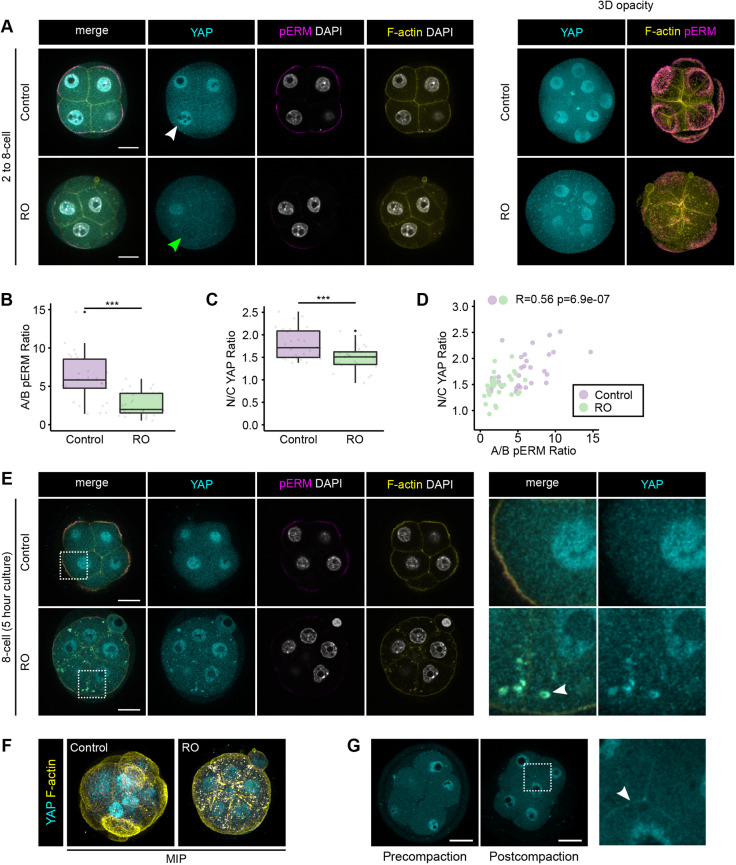
**Biochemical changes occurring during compaction are required for the nuclear accumulation of YAP in a subset of blastomeres from the two- to eight-cell stage.** (A) Representative images of DMSO- and RO-treated embryos grown *in vitro* from the two- to the eight-cell stage immunostained for YAP and pERM. F-actin and nuclei were visualised using Phalloidin and DAPI, respectively. White arrowhead indicates a nucleus with high levels of YAP, whereas green arrowhead indicates a nucleus with low levels of YAP. Right panel shows three-dimensional opacity renderings of corresponding embryos. Scale bars: 20 µm. (B) Boxplot showing the proportion of pERM at the apical membrane in control (*n*=4 embryos) and RO-treated (*n*=4 embryos) embryos. (C) Boxplot showing the proportion of YAP in the nucleus in control and RO-treated embryos. ***P<0.001 (Kruskal–Wallis test). (D) Plot showing the relationship between the proportion of pERM at the apical membrane and N/C YAP ratio in control and RO-treated embryos (Spearman, *R*=0.56, *P*=6.9×10^−7^). (E) Representative images of embryos cultured for 5 h at the eight-cell stage in the presence of either DMSO or RO and subsequently immunostained for YAP and pERM. F-actin and nuclei were visualised using Phalloidin and DAPI, respectively. Right panel shows magnification of the areas surrounded by dashed outlines. The white arrowhead indicates cytoplasmic puncta of F-actin and YAP. Scale bars: 20 µm. (F) Maximum intensity projections of embryos shown in E. (G) Representative images of pre- and postcompaction eight-cell embryos immunostained for YAP and showing blastomeres with comparable N/C YAP ratios. The panel on the right shows a high-magnification image of the boxed area. The white arrowhead points at YAP localised at cell-cell junctions.

Embryos treated with inhibitor from the two- to the eight-cell stage not only exhibited a reduced proportion of apical to basolateral pERM (A/B pERM ratio) but were also unable to form apical actin rings, consistent with delayed or perturbed organisation of the apical domain (Fig. 4A,B). RO treatment was also accompanied by a markedly reduced N/C YAP ratio (Fig. 4A,C). Moreover, when we plotted the correlation between the N/C YAP ratio and the A/B pERM ratio, we could detect a moderate positive correlation (*R*=0.56, *P*=6.9×10^−7^), suggesting that the N/C YAP ratio was reduced by an amount proportional to pERM inhibition (Fig. 4D). In order to understand further how the RO compound impacted blastomeres from the two- to the eight-cell stage, we examined the clusters to which RO-treated and control blastomeres belonged. Interestingly, we found a similar proportion of cells between control (13/26 cells, 50%) and RO-treated embryos (15/29 cells, 52%) outside the undefined cluster (Fig. S4A,B), suggesting that the RO inhibitor did not prevent blastomeres from maturing out of the undefined cluster. However, outside the undefined cluster, we could observe two differences between RO-treated and control blastomeres. An increased number of RO-treated blastomeres could be found in the inside-like cluster (5/29 cells, 17.2% of RO-treated blastomeres and 0/26, 0% of control blastomeres). In the outside-like cluster, RO-treated blastomeres exhibited lower N/C YAP ratios in comparison to DMSO-treated blastomeres. This suggests that disrupting the organisation of the apical domain using RO prevented the N/C YAP ratio from increasing in blastomeres with a high proportion of exposed surface area. These results are therefore in agreement with a potential role for the apical domain (as opposed to the maturation of blastomeres out of the undefined cluster) in establishing the relationship between the N/C YAP ratio and the proportion of exposed surface area of blastomeres during compaction.

To test the importance of the actin cytoskeleton, we also treated embryos from the two- to eight-cell stage with cytochalasin D (CCD). CCD treatments resulted in a low proportion of blastomeres being allocated outside the undefined cluster (4/21, 19%) in comparison to DMSO controls (14/25, 56%), suggesting that CCD-treated blastomeres were unable to mature out of the undefined cluster (Fig. S4C,D). Given that the majority of CCD-treated blastomeres were also unable to polarise (Fig. S4E), it remains unclear which precise biological process controls the transition of blastomeres from an undefined state to a state in which the proportion of exposed surface and the N/C YAP ratio are correlated.

Shorter (5 h) treatment with RO inhibitor starting later, at the eight-cell stage, did not result in a significant decrease in nuclear YAP. One potential explanation for this reduced effect on the N/C YAP ratio could be that PKC activity started before the treatment, giving enough time for YAP to accumulate in the nuclei of blastomeres with a high proportion of exposed surface area (Fig. 4E). Interestingly, the 5 h RO treatment resulted in both YAP and F-actin accumulating and colocalising in small cytoplasmic aggregates (Fig. 4E,F). High-resolution imaging showed that even in unmanipulated embryos, YAP can be detected at cell-cell junctions (Fig. 4G; Fig. S4F), suggesting that YAP can interact, most probably in an indirect manner, with cortical F-actin. Collectively, our data suggest that the organisation of the F-actin cytoskeleton and the apical domain around the time of compaction is required for the nuclear accumulation of YAP observed in a subset of blastomeres with a high proportion of exposed surface area, suggesting that this might represent an important step in allowing blastomeres to develop the ability to sense the proportion of their surface that is exposed and, ultimately, their position within the preimplantation embryo.

### Manipulation of embryo shape alters blastomere fate and reveals position sensing at the eight-cell stage

**Fig. 5.**
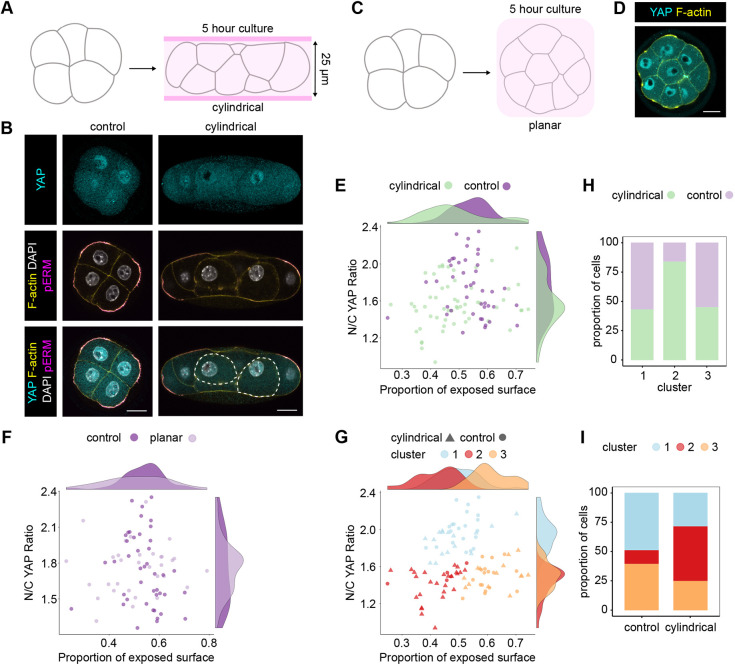
**Manipulation of embryo shape reveals position sensing at the eight-cell stage.** (A) Diagram representing the experimental design to obtain cylindrical embryos. Eight-cell embryos were inserted into channels 25 µm in diameter and cultured for 5 h. (B) Representative images of control and cylindrical eight-cell embryos immunostained for YAP and pERM. F-actin and nuclei were visualised using Phalloidin and DAPI, respectively. Scale bars: 20 µm. (C) Diagram representing the experimental design to obtain planar embryos. Eight-cell embryos were covered with a hydrogel sheet and cultured under confinement for 5 h. (D) Representative image of a planar eight-cell embryo immunostained for YAP. F-actin was visualised using Phalloidin. Scale bar: 20 µm. (E) Plot showing the proportion of exposed surface and N/C YAP ratio in control (*n*=6 embryos) and cylindrical (*n*=7 embryos) embryos. Marginal density plots for control and cylindrical embryos, on the sides of the graph, show a shift in both the proportion of exposed surface and the N/C YAP ratio in blastomeres from cylindrical embryos. (F) Plot showing the proportion of exposed surface and the N/C YAP ratio in control (*n*=6 embryos) and planar (*n*=4 embryos) embryos. Note the absence of changes in the proportion of exposed surface and N/C YAP ratio in the marginal density plots. (G) Representation of the proportion of exposed surface and N/C YAP ratio in blastomeres from control and cylindrical embryos and the different clusters obtained by hierarchical clustering. (H) Bar graph representing the proportion of blastomeres from control or cylindrical embryos in each cluster. (I) Proportion of blastomeres from each cluster in control and cylindrical embryos.

Our results so far suggest the hypothesis that blastomeres use the proportion of their surface area that is exposed to the outside as a measure of their position within the embryo to modulate the subcellular localisation of YAP. A prediction of this hypothesis is that altering the proportion of surface area exposed will lead to a corresponding alteration in the N/C YAP ratio. To test this prediction without resorting to dissociating individual blastomeres, which might perturb signals between contacting blastomeres, we developed a non-invasive approach to alter the shape and position of cells in the precompaction embryo. Our aim was to modulate the distribution of the proportion of exposed surface area amongst blastomeres within each embryo in order to analyse the consequences on the N/C YAP ratio. To this end, we used biocompatible hydrogels to create channels of consistent diameters, in which to culture embryos. This physical constraint forced embryos to adopt a cylindrical shape, leading to blastomeres becoming relatively more embedded within the embryos (Fig. 5A,B; Movie 2). As a second approach to modify embryo geometry, we cultured embryos between sheets of hydrogels, which resulted in planar embryos with all blastomeres localised within a single plane and an increased spread in the distribution of exposed surface area (Fig. 5C,D; Movie 3). These approaches allowed us to maintain polarity and adhesion between blastomeres.

When cultured within channels for 5 h (Fig. 5B), the majority of blastomeres exhibited a reduction in the proportion of surface area exposed. This resulted in overall lower values for the proportion of exposed surface in comparison to controls (Fig. 5E; Fig. S5A). Consistent with the predictions of the hypothesis, shifting the distribution of the proportion of exposed surface in this way was accompanied by a significant decrease in the median N/C YAP ratio in blastomeres (Fig. 5E; Fig. S5B). In contrast to the cylindrical embryos, in the planar embryos, both the median N/C YAP ratio and the proportion of exposed surface area remained close to the respective values from controls (Fig. 5F; Fig. S5A,B), supporting the view that it is specifically changes in the distribution of exposed surface area (rather than other factors related to the compression of embryos within hydrogels) that led to changes in the YAP ratio. The A/J ratio was also significantly shifted in cylindrical embryos, raising the potential for this being the factor determining YAP localisation. However, arguing against this, the A/J ratio was also shifted in a similar manner in planar embryos, in which there was no overall change in the distribution of N/C YAP (Fig. S5C).

To test in an unbiased manner whether cylindrical embryos were able to generate a new population of cells simultaneously characterised by a lower proportion of exposed surface and a reduced N/C YAP ratio, we performed hierarchical clustering of these blastomeres on the basis of the N/C YAP ratio and the proportion of exposed cell surface area. This resulted in three clusters (Fig. 5G; Fig. S5D). Strikingly, cluster 2, containing blastomeres with the lowest proportion of exposed surface area and N/C YAP ratio, was predominantly composed (83.9%) of cells from cylindrical embryos (Fig. 5H), and close to half (46.4%) of the blastomeres from cylindrical embryos belonged to this cluster (Fig. 5I). When considering blastomeres from clusters 1 and 2 (showing properties similar to the outside-like and inside-like clusters, respectively), the proportion of exposed surface area had the highest correlation coefficient with the N/C YAP ratio, confirming our results in unmanipulated embryos (Fig. S5E). Together, these results suggest that changes to the proportion of exposed blastomere surface area might result in changes in the N/C YAP ratio. This tight relationship indicates that blastomeres from as early as the eight-cell stage are able to sense and respond rapidly to subtle changes in their position to modulate YAP localisation.

Although blastomeres from cylindrical embryos showed an overall reduction in exposed surface area, they were prevented from becoming completely internalised by the cylindrical confinement. We took advantage of this to test our previous observations suggested that postcompaction, eight-cell blastomeres are on a TE trajectory (Fig. 3B,C). When eight-cell embryos were grown inside channels until the 16-cell stage, in comparison to the controls, fewer cells were internalised and fewer excluded YAP from the nucleus, suggesting that fewer ICM cells were formed (Fig. S5F). To test whether this was indeed the case, we cultured embryos from the eight- to the 32-cell stage in channels within polyacrylamide of varying stiffness (see Materials and Methods for details) to subject them to either moderate or extreme confinement. We then stained the embryos for CDX2 to assay blastomere fate. In embryos under extreme confinement, all cells were forced to occupy the outer surface, and it was possible to create embryos consisting solely of CDX2^+^ cells (Fig. S5G). This effect was titratable; under milder compression, blastomeres managed to move inside and downregulate CDX2, but their numbers decreased with increasing tissue deformation (Fig. S5H). These experiments show that simply by preventing inside cells from arising, it is possible to derive embryos made exclusively of CDX2^+^ blastomeres, consistent with eight-cell blastomeres existing on a TE trajectory and with blastomere position and internalisation being the main driving force behind the emergence of the ICM lineage.

## DISCUSSION

**Fig. 6.**
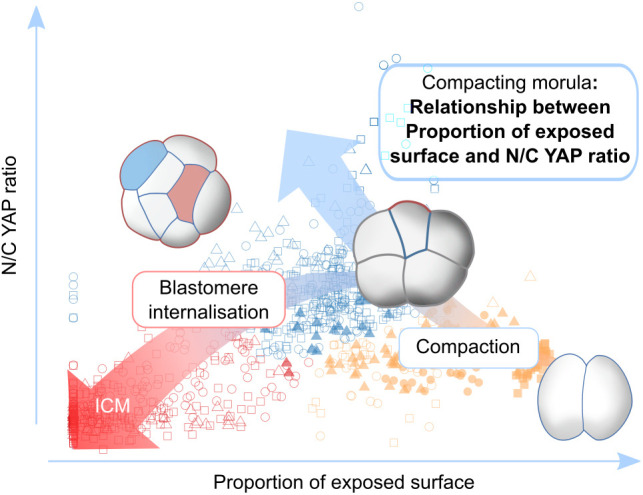
**Summary diagram.** A relationship between the proportion of exposed surface and the N/C YAP ratio emerges in blastomeres during compaction. Our results suggest that blastomeres are on a TE trajectory from the the two- to the eight-cell stage. Subsequently, blastomere internalisation drives the emergence of the ICM lineage.

We have discovered that, as early as during compaction at the eight-cell stage, blastomeres start to exhibit a close relationship between the proportion of their surface exposed and the proportion of YAP in their nucleus and cytoplasm (Fig. 6). This suggests that after polarisation, blastomeres can sense the proportion of their surface area exposed within the embryos and transfer this information to the nucleus by modulating the subcellular localisation of YAP.

To demonstrate the position-sensing ability of blastomeres within the embryo, we used a new approach that, as opposed to the more traditional use of chimeras or dissociated blastomeres, does not alter the number of cells or the structural integrity of embryos (Leonavicius et al., 2018). By inserting embryos within cylindrical channels of defined diameter, we were able to alter embryo shape and the relative position of blastomeres. Using this method, we also showed that blastomeres of the eight-cell embryo are on a TE trajectory because, when blastomere internalisation was prevented, TE fate was favoured. This confirms the crucial role of blastomere internalisation in the emergence of the ICM lineage.

After compaction, in the next round of cell divisions, the first inner cells will be formed. However, it remains unclear precisely how this is achieved (White et al., 2018). It has been suggested that cell fate decisions operate differently from the eight- to 16-cell stage (Anani et al., 2014) and from the 16- to 32-cell stage, respectively (Hirate et al., 2013), with asymmetric cell divisions making a more important contribution during the eight- to 16-cell transition. However, clear asymmetric divisions rarely occur (Samarage et al., 2015), and in many cases, the apical domain seems to disassemble when blastomeres divide before being re-established *de novo* after cytokinesis (Zenker et al., 2018). Our results indicate that blastomere position within the embryo, as a result of either the mode of division or the movement of cells, is ultimately what defines cell fate, consistent with cell division angle not determining fate (Watanabe et al., 2014). Instead, we propose that changes of global embryo geometry via cell internalisation drive the formation of the ICM, which is consistent with the important role of apical constrictions in cell internalisation to form the ICM (Anani et al., 2014; Samarage et al., 2015). Given that we see a relationship between the relative amounts of YAP in the nucleus and the proportion of exposed surface, it raises the question of whether nuclear YAP levels themselves can influence cell internalisation. It seems plausible that after cell division at the eight-cell stage, owing to increased cell crowding, some cells exhibit a lower proportion of exposed surface by chance, leading to lower relative nuclear YAP levels. It is tempting to speculate that this triggers a positive feedback loop between the reduced proportion of exposed surface and the progressive nuclear exclusion of YAP, leading to cell internalisation. This hypothesis is supported by work in *Drosophila*, showing the importance of the Hippo pathway in regulating apical domain size and apical complexes (Genevet et al., 2009; Hamaratoglu et al., 2009). Ultimately, identifying the genes that are regulated by the Hippo pathway during preimplantation development will help to shed light on these challenging questions. Together, our results highlight the truly regulatory nature of the mouse preimplantation embryo to adapt and integrate global geometry changes into cell fate decisions.

What mechanism might blastomeres use to measure the proportion of their surface that is exposed to the exterior? Polarisation at the eight-cell stage is the first event generating asymmetries within blastomeres from a structural point of view (Ducibella et al., 1977; Louvet et al., 1996; Vinot et al., 2005; Ziomek and Johnson, 1980). Recent work has advanced our understanding of how the process of polarisation might be coupled to the morphogenetic events of compaction (Zhu et al., 2017). The actomyosin contractility machinery that mediates compaction is recruited to cell contact-free membrane in a chain of events initiated by phospholipase C (PLC) that ultimately leads to the establishment of apicobasal polarity. Importantly, the organisation of the contractility machinery at the cell cortex enables the generation of the forces driving compaction (Maître et al., 2015). This body of work highlights one of the major outcomes of compaction: the creation of two different membrane domains, apical and basolateral, with different organisation, protein complexes and contractile properties. Several of our observations suggest that YAP might be able to localise to these different membrane domains. First, we can detect YAP at cell-cell junctions, along the basolateral domain, in eight-cell embryos. Second, when the RO compound was used in eight-cell embryos, YAP accumulated in cytoplasmic puncta, where it colocalised with F-actin. Given that PKC inhibition reduces the phosphorylation of apical ERM and disrupts apical F-actin organisation, we speculate that YAP can interact indirectly with apical actin. Together, these observations suggest a mechanism whereby YAP localised at the apical and basolateral domains is regulated differently. The balance between the inhibitory signal at the basolateral membrane and the activating signal at the apical membrane might provide the cell with a measure of the proportion of exposed cell surface area. Hippo interactome studies reveal that YAP interacts, directly or indirectly, with various components of not only the apicobasal polarity complexes but also planar polarity molecules and other proteins associated with the plasma membrane (Couzens et al., 2013; Hauri et al., 2013; Wang et al., 2014). It will therefore be important to determine which of those play a role in the preimplantation embryo to regulate the subcellular localisation of YAP in the context of position sensing.

## MATERIALS AND METHODS

### Mouse husbandry and embryo collection

Mice were housed in a 12 h dark, 12 h light cycle. CD1 females (Charles River, UK) were crossed with C57BL/6J males (in house) to obtain stage-specific embryos. Noon of the day of finding the mating plug was defined as 0.5 days postcoitum. Most experiments were performed using flushed embryos from natural mating. For superovulations, 8-week-old CD1 females were given intraperitoneal injections of 5 IU of pregnant mare serum gonadotropin (PMSG) followed by 5 IU of human chorionic gonadotropin (hCG) 48 h later and were mated with C57BL/6J males. Embryos were flushed using M2 medium at the indicated stages (Sigma M7167). All experimental procedures complied with Home Office regulations (project licence 30/3420) and were compliant with the UK Animals (Scientific Procedures) Act 1986 and approved by the local Biological Services Ethical Review Process.

### Human embryo collection

Human embryos were donated from patients attending Oxford Fertility, with approval from the Human Fertilization and Embryology Authority (centre 0035, project RO198) and the Oxfordshire Research Ethics Committee (reference number 14/SC/0011). Informed consent was attained from all patients. Embryos were fixed in 4% paraformaldehyde, washed twice and kept in PBS containing 2% bovine serum albumin (2% PBS-BSA) at 4°C until they were used for immunostaining.

### Wholemount immunostaining

After fixation with 4% paraformaldehyde (PFA) for 15 min, embryos were washed twice in 2% PBS-BSA. Embryos were then permeabilized with PBS containing 0.25% Triton X-100 (PBS-T) for 15 min and subjected to two washes in 2% PBS-BSA at room temperature. Embryos were then placed in blocking solution for 1 h (3% BSA, 2.5% donkey serum in PBS containing 0.1% Tween). Incubation with primary antibodies took place overnight at 4°C in a humidified chamber. The following primary antibodies were used in this study and diluted in blocking solution at the indicated concentrations: mouse-anti-YAP (1:100, Santa Cruz Biotechnology, sc-101199), rat-anti-E-cadherin (1:100, Sigma, U3254), rabbit anti-pERM (1:200, Cell Signaling, 3141), rabbit anti-CDX2 (1:100, Cell Signaling, 3977) and rabbit anti-Par6b (1:100, Santa Cruz Biotechnology, sc-67393). The next day, embryos were washed three times in 2% PBS-BSA for 15-20 min. Embryos were then incubated with secondary antibodies and Phalloidin (1:100 in blocking solution for 1 h). The following reagents were used (1:100): Alexa fluor 555 donkey-anti-mouse (Invitrogen, A-31570), Alexa fluor 647 goat-anti-rat (Invitrogen, A-21247), Alexa fluor 488 donkey-anti-rabbit (Invitrogen, A21206), Phalloidin-Atto 488 (Sigma, 49409) and Phalloidin-Atto 647N (Sigma, 65906). After another three washes of 15-20 min in 2% PBS-BSA, the embryos were mounted in eight-well chambers in droplets consisting of 0.5 µl Vectashield with DAPI (Vector Laboratories) and 0.5 µl 2% PBS-BSA. Embryos were transferred between solutions by mouth-pipetting. All incubations took place at room temperature, unless stated otherwise. After mounting, the embryos were kept in the dark at 4°C until they were imaged.

### Confocal microscopy

Embryos were imaged on a Zeiss LSM 880 confocal microscope, using a C-Apochromat ×40/1.2 W Korr M27 water immersion objective. Laser excitation wavelengths were 405, 488, 561 and 633 nm, depending on the specific fluorophore. Embryos were imaged using a ×1.5 zoom at a resolution of 512×512 pixels, and eight-bit depth *z*-stacks of entire embryos were acquired at 1 µm intervals using nonsaturating scan parameters.

### Embryo culture

Embryos were cultured in organ culture dishes in 500 µl pre-equilibrated Evolve medium (Zenith Biotech) at 37°C in air enriched with 5% CO_2_ for the indicated amount of time. The PKC inhibitor Ro-31-8220 (RO; Calbiochem, 19-163) was diluted in DMSO and used at 2.5 µM (1:2000 dilution). Cytochalasin D (CCD; Sigma-Aldrich, C8273) was used at 0.5 µg/ml. The same amount of DMSO was used in control cultures, and embryos were either cultured from the two- to the eight-cell stage or for 5 h starting at the eight-cell stage.

For cylindrical embryo cultures, channels were formed by casting a 5% (which corresponded to ∼4.2 kPa stiffness) acrylamide hydrogel (containing 39:1 bisacrylamide) around 25 µm wires within the confinement of a two-part mould (10 mm×10 mm×1 mm). In milder compression experiments, the amount of acrylamide/bisacrylamide was reduced to create softer gels of ∼3.5 kPa stiffness (Tse and Engler, 2010). Ammonium persulphate (0.1%) and tetramethylethylenediamine (TEMED) (1%) were added to polymerize polyacrylamide. The wires were then removed to form cylindrical cavities within hydrogel pieces, which were cut to ∼3 mm×3 mm×1 mm blocks for easier manipulation during embryo insertion. The hydrogels were washed carefully and equilibrated in embryo culture media at 37°C and in air enriched with 5% CO_2_ overnight. The embryos were then inserted into the channels using a glass capillary with a diameter slightly larger than the embryo itself. It was used to stretch the hydrogel channel before injecting the embryos and letting the channels relax and deform the embryos. Cell viability in channels had been assessed previously, without any noticeable difference in control embryos (Leonavicius et al., 2018).

For planar deformations, embryos were overlaid with a sheet of 5% acrylamide hydrogel. Excess medium around the embryo was withdrawn with a micropipette to ensure that the acrylamide sheet would force the embryo to adopt a planar configuration. At the end of the experiments, embryos were fixed inside the hydrogels with 4% PFA for 20 min. Once fixed, embryos were then removed from the hydrogel channels and immunostained in parallel to the controls.

### Segmentation and image analysis

Manual segmentation of confocal data was done using Imaris v.6.3 (Bitplane). Outlines of the cell and nucleus were drawn using a Wacom Cintique 21UX tablet display to create a three-dimensional surface of each blastomere membrane and nucleus using the contour surface function. Information about geometry (sphericity, total surface area, volume, oblate and prolate) and signal intensity within each compartment could then be exported. Information about blastomere exposed, contact and junctional surface areas was obtained by considering surface proximities and was automated using a software package developed in house (Javali et al., 2017; Leonavicius et al., 2020). The signal intensity around these defined membrane domains could then be extracted. Dividing cells were excluded from the analysis, because their geometry parameters were widely different from non-dividing cells and their nuclear envelope disassembled. Imaris files of segmented embryos will be made available on request.

We used the proportion of exposed surface area (and its converse, the proportion of contact area) as a measure of whether a blastomere is embedded within the embryo or is on the surface. The apical surface can be domed or flat, potentially indicative of, or leading to, increased or decreased tensions at the apical junctions. As an estimate of this, we also assessed the extent to which the apical domain was protruding out of the embryo by calculating the ratio between the apical surface area and the apical junction interface area (A/J ratio). The apical junctional interface was represented by a narrow band and was therefore expressed as an area, resulting in the A/J ratio being dimensionless.

Using the approaches above, we categorised the following parameters as relating to blastomere shape: sphericity; oblateness; prolateness; and volume. We categorised the following parameters as relating to blastomere position: absolute and proportion of contact area; absolute and proportion of exposed area; absolute and proportion of junctional area; and ratio between apical and junctional area.

### Clustering and statistical analysis

Figures and diagrams were assembled and created using the free and open source software, Inkscape and Krita. All statistics and graphs were done using RStudio and R. Graphs were produced using several packages, including ggplot2 and ggpubr. For statistical analysis, the normality of the data was first assessed using visualisation tools and statistical tests (Shapiro–Wilk normality test). When the data were normally distributed, we used analysis of variance (ANOVA), followed by post hoc comparisons using Tukey's HSD test when comparing more than two conditions. Otherwise, the Kruskal–Wallis test was used, followed by post hoc comparison using Dunn's test. To test the correlation between two variables, Spearman's method was used when the two variables were not normally distributed. The Corrplot package was used to create a correlation matrix of the different variables in the preimplantation dataset. To define the N/C YAP ratio threshold between cells with high and low YAP ratio by *k*-means clustering, the data were standardized, and distance measures were obtained using the Euclidean method. For hierarchical clustering analysis, the dynamicTreeCut function was used to determine the ideal number of clusters. The variables were scaled, and the distance matrix was produced using the Euclidean method. Ward's method was used to perform the clustering.

## Supplementary Material

Supplementary information
