# Supplementary material for: Establishment of a relationship between blastomere geometry and YAP localisation during compaction
Source: Development. 2020 Oct 9;147(19):dev189449. doi: 10.1242/dev.189449 (PMC7561472; doi:10.1242/dev.189449)
Supplement: Supplementary information [file develop-147-189449-s1.pdf]

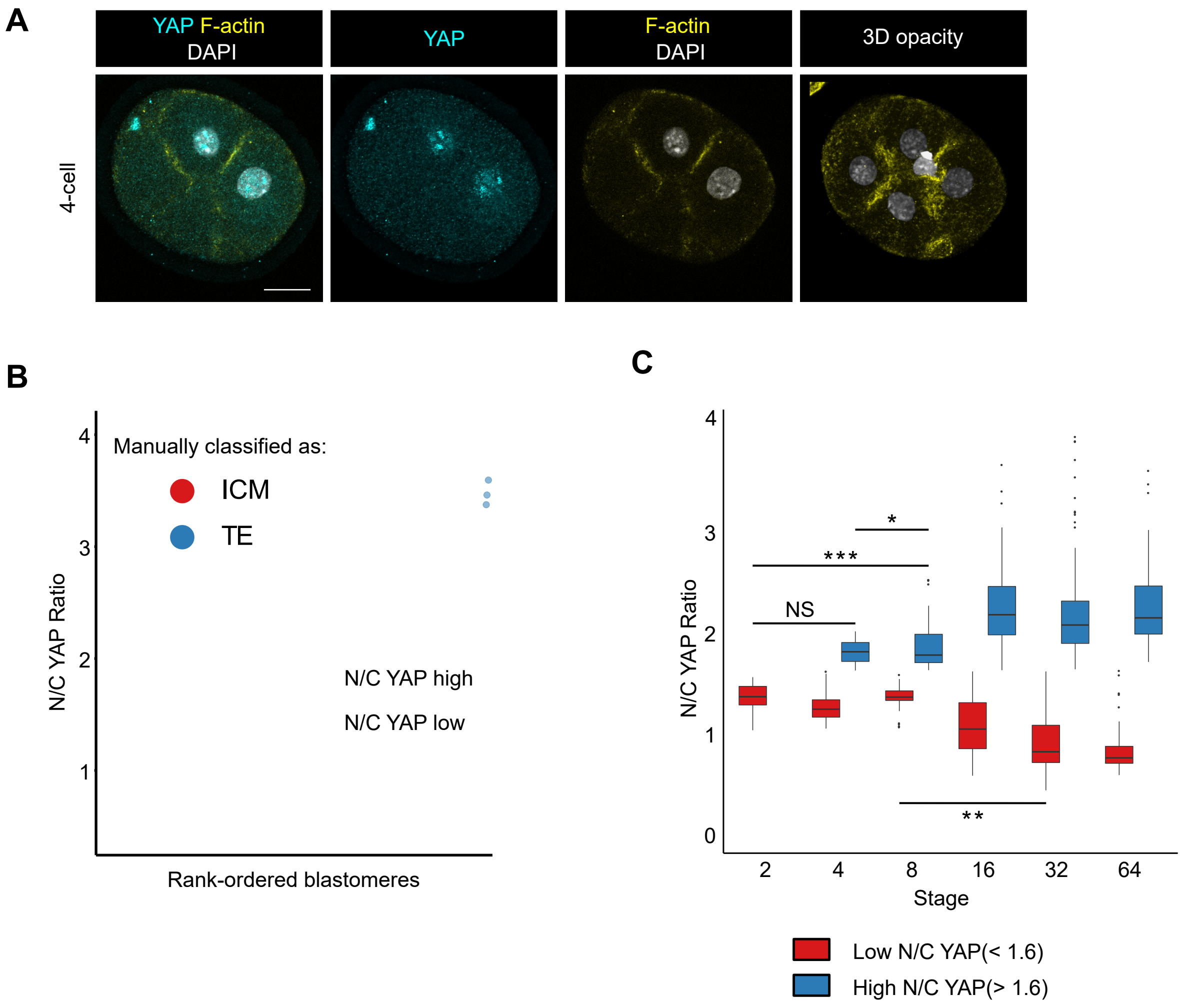

Fig. S1. Unbiased separation of blastomeres with low and high N/C YAP ratio using K-means clustering. A. To evaluate the validity of the threshold separating high and low N/C YAP ratios obtained using the K-means algorithm on all blastomeres from the 2- to 64-cell stage, blastomeres from 32- and 64-cell embryos were ranked by ascending N/C YAP ratio and manually categorised as ICM (red) or TE (blue) depending on their localisation within those embryos. The dashed line represents the threshold value separating high and low N/C YAP ratios, as defined by K-means algorithm. B. Blastomeres with low and high N/C YAP ratio across developmental stages. Blastomeres were classified as exhibiting either high (>1.6) or low (<1.6) N/C YAP ratio based on K-means algorithm to separate them into two populations in an unbiased manner. NS: not significant. * p<0.05, ** p<0.01, *** p<0.001 (Kruskal Wallis test followed by Dunn's test).

Development • Supplementary information

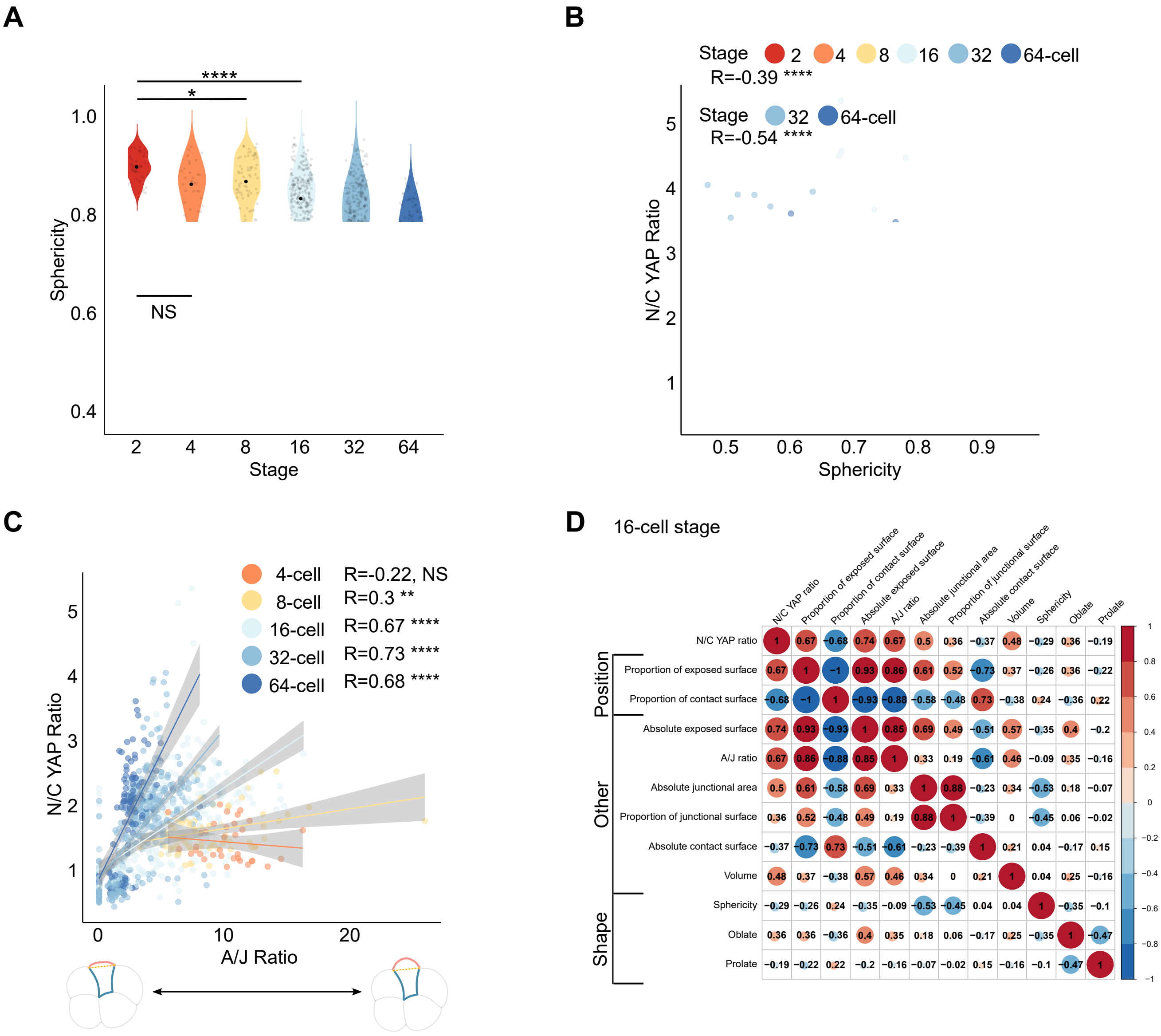

Fig. S2. Sphericity is a poor predictor of N/C YAP ratio. A. Sphericity across developmental stages. Median sphericity for each developmental stage is represented as a black dot (Kruskal Wallis test followed by Dunn's test). B. Correlation analysis between sphericity (shape) and N/C YAP ratio at the indicated stages of development (Spearman). C. Correlation analysis between A/J ratio (shape of the apical domain) and N/C YAP ratio at the indicated stages of development (Spearman). NS: not significant. * $p<0.05$, ** $p<0.01$, **** $p<0.0001$. D. Correlation matrix between N/C YAP ratio and geometric characteristics of individual blastomeres at the 16-cell stage. The value of the correlation coefficient (Spearman) between two variables is indicated and also represented by the size and colour of the circles.

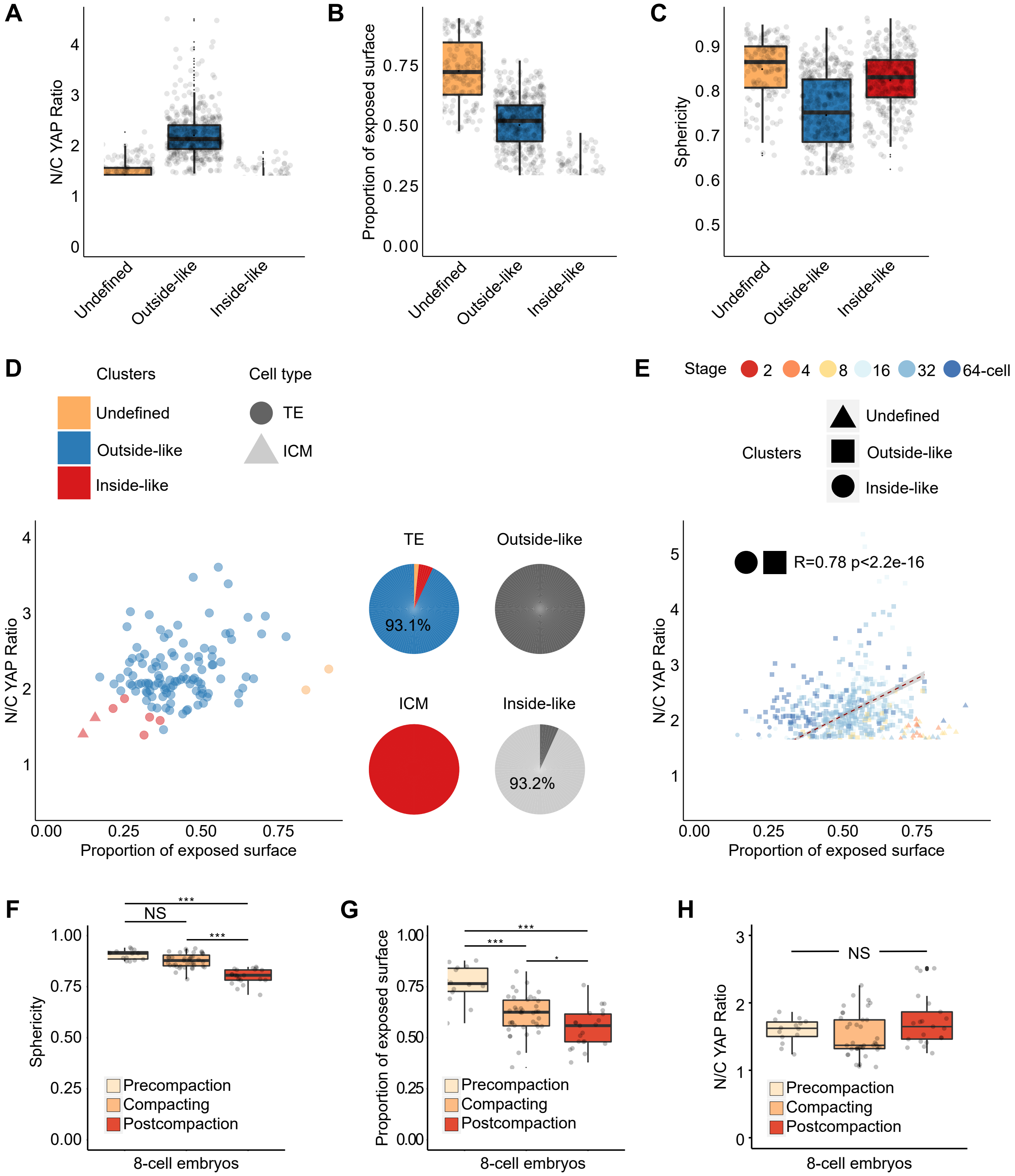

Fig. S3. Validation of clusters obtained by hierarchical clustering. A. N/C YAP ratio of individual blastomeres across development in undefined, outside-like and inside-like clusters B. Proportion of exposed surface of individual blastomeres in undefined, outside-like and inside-like clusters. C. Sphericity of individual blastomeres in undefined, outside-like and inside-like clusters. D. Unsupervised hierarchical clustering of blastomeres from 32- and 64-cell embryo (see results for description of the three cluster types). ICM (light grey) or TE (dark grey) identity was manually assigned to blastomeres based on their position to validate the clustering. The pie charts display the proportion of different types of blastomeres that make up each category. All Outside-like and the majority of Inside-like blastomeres were TE and ICM respectively. E. Scatter plot of blastomere proportion of exposed surface and N/C YAP ratio in blastomeres across development (dot colour represents stages) with information on the clusters they belong to (dot shape). F, G and H. Sphericity, proportion of exposed surface area and N/C YAP ratio of individual blastomeres in precompaction (n=2 embryos), compacting (n=5 embryos) and postcompaction (n=3 embryos) embryos. NS: not significant. *** $p<0.001$, * $p<0.05$ (Kruskal Wallis test followed by Dunn's test).

Development • Supplementary information

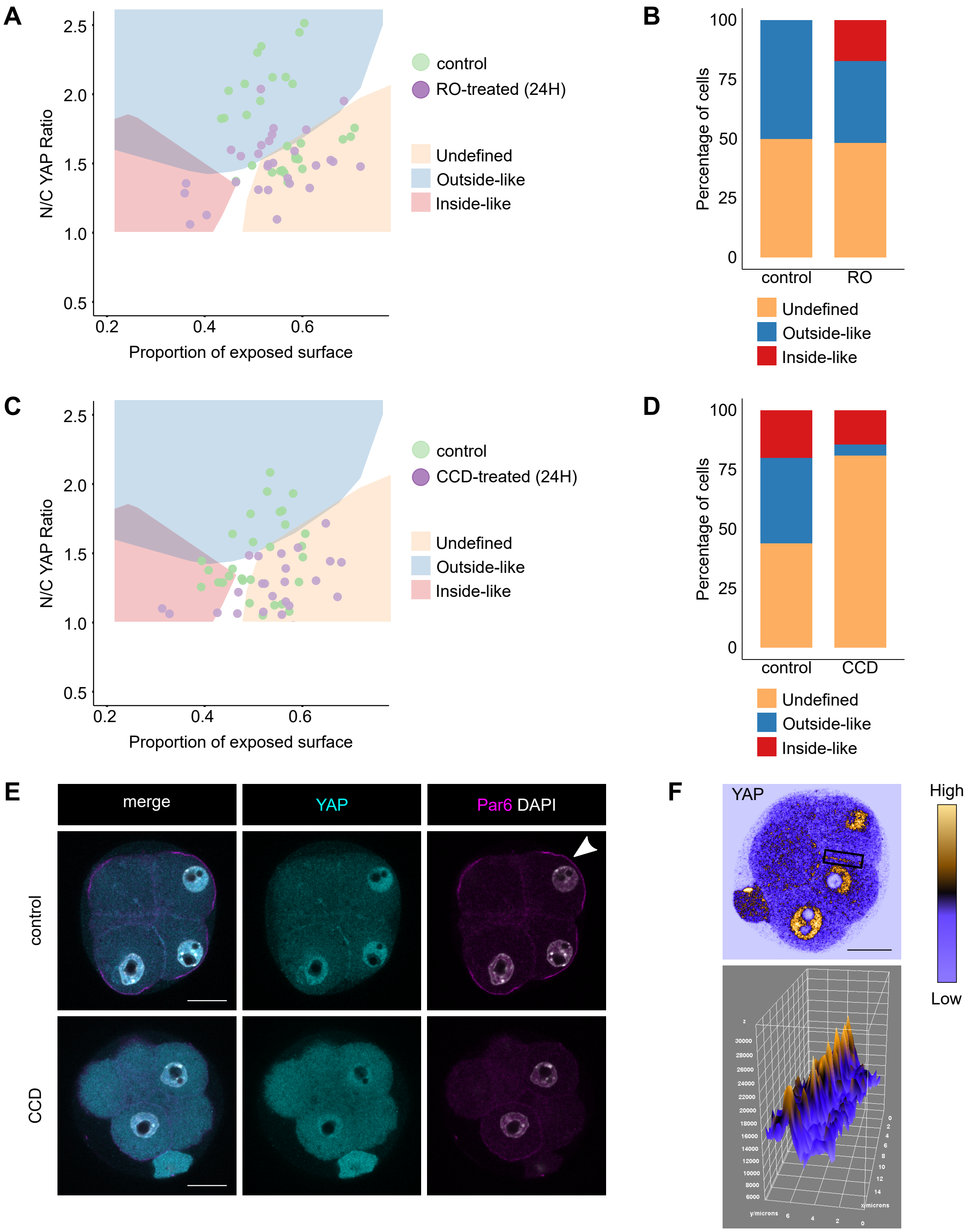

Fig. S4. Biochemical changes occuring during compaction are essential to establish the relationship between proportion of exposed surface area and N/C YAP ratio. A. Scatter plot of blastomere proportion of exposed surface and N/C YAP ratio of control (n=4 embryos) and RO-treated (n=4 embryos) embryos. The areas covered by the different clusters are indicated in the graph. B. Proportion of blastomeres belonging to each cluster in control and RO-treated embryos. C. Scatter plot of blastomere proportion of exposed surface and N/C YAP ratio of control (n=4 embryos) and cytochalasin D-treated (n=3 embryos, CCD) embryos. The areas covered by the different clusters are indicated in the graph. D. Proportion of blastomeres belonging to each cluster in control (n=4 embryos) and CCD-treated (n=3 embryos) embryos. E. Representative images of embryos cultured from the 2- to the 8-cell stage in the presence of either DMSO or CCD and subsequently immunostained for YAP and Par6. F-actin and nuclei were visualised with Phalloidin and DAPI respectively. The white arrowhead points to apically localised Par6. F. Representative image of an 8-cell embryo immunostained for YAP. The bottom panel represents the signal intensity profile of the area outlined by a black box. Scale bar: 20 µm.

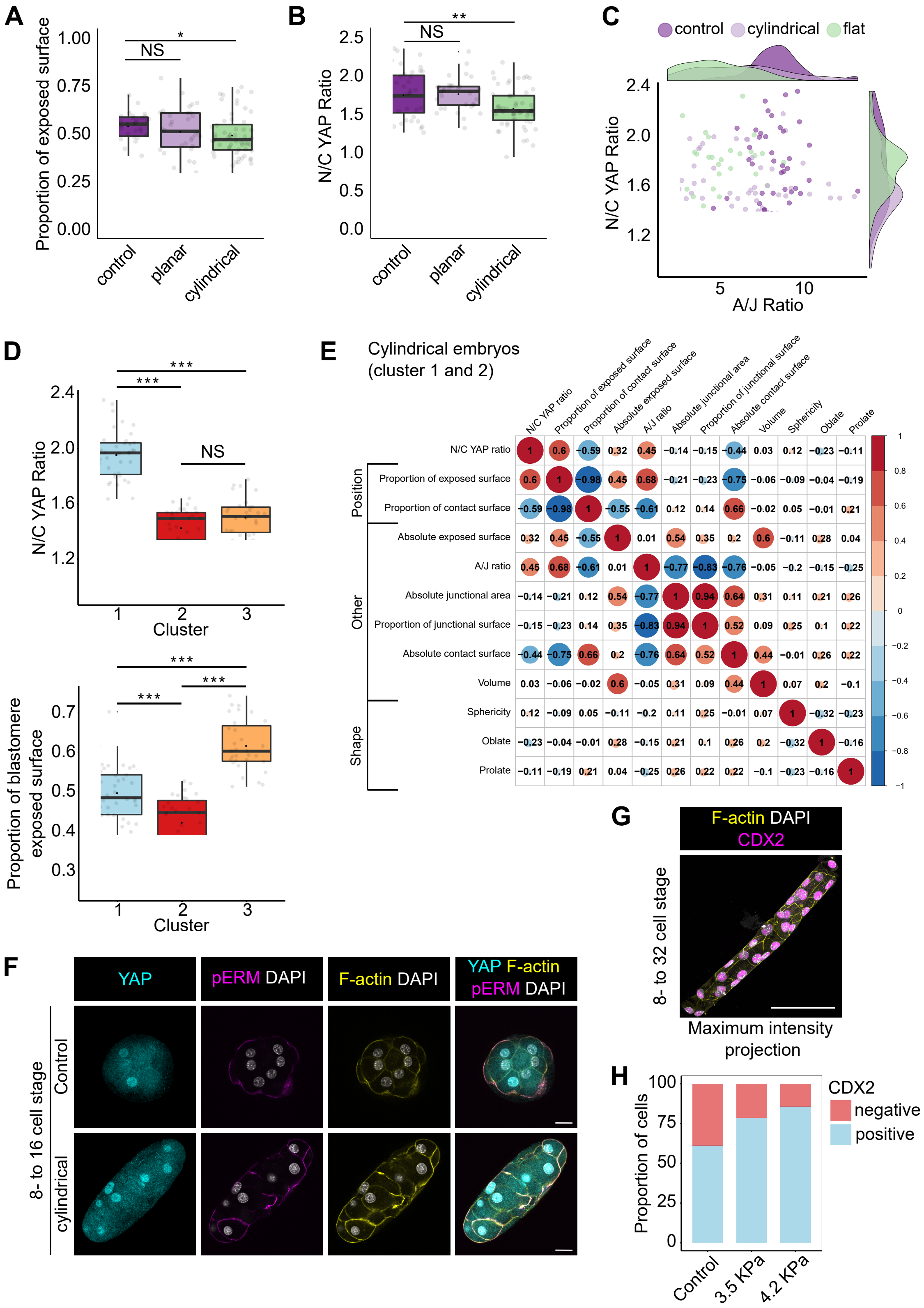

Fig. S5. Effect of embryo shape manipulation at the 8-cell stage on embryo and blastomere geometry. A. Boxplot of the proportion of exposed surface in control, planar and cylindrical embryos. B. Boxplot of N/C YAP ratio in control, planar and cylindrical embryos. C. Plot showing A/J ratio and N/C YAP ratio in control (n=6 embryos), planar (n=4 embryos) and cylindrical (n=7 embryos) embryos. Marginal density plots on the sides of the graph, show a shif of A/J ratio in blastomeres from both cylindrical and planar embryos. D. N/C YAP ratio and proportion of exposed surface in the different clusters obtained from control and cylindrical embryos based on N/C YAP ratio and the proportion of exposed surface area. E. Correlation matrix between N/C YAP ratio and geometric characteristics of individual blastomeres belonging to cluster 1 and 2 from cylindrical embryos. F. Representative images of 8-cell embryos grown to the 16-cell stage in control conditions or in 25 µm channels and subsequently immunostained using antibodies against YAP and pERM. F-actin and nuclei were visualised using Phalloidin and DAPI respectively. Scale bar: 20 µm. G. An example of an 8-cell embryo grown until the 32-cell stage in a 25 µm channel, in which all cells are forced to be on the “outside”. Note how all cells are CDX2 positive. Scale bar: 50 µm. H. Quantification of CDX2-positive and negative cells in 16-cell embryos following two different levels of compression at the 8-cell stage, using gels of 3.5 and 4.2 KPa in stiffness respectively. Control: 121 cells; 3.5 KPa: 98 cells; 4.2 KPa: 84 cells. NS: not significant. * $p<0.05$, ** $p<0.01$, *** $p<0.001$ (D, Kruskal Wallis test followed by Dunn’s test; A, B, ANOVA followed by the Tukey HSD test).
